# Clinical benefit and predictors of response to momelotinib after ruxolitinib failure: A cooperative real‐world study

**DOI:** 10.1002/cncr.70457

**Published:** 2026-05-12

**Authors:** Francesca Palandri, Monia Marchetti, Elena M. Elli, Alessandro Isidori, Ilaria Gianesello, Chiara Sartor, Novella Pugliese, Maria Di Perna, Erika Morsia, Susanna Gallo, Federico Itri, Vittorio Montefusco, Elena Crisà, Annalisa Biagi, Eloise Beggiato, Olga Mulas, Giulia Benevolo, Elisabetta Abruzzese, Marco Santoro, Alessandra Iurlo, Elisabetta Calistri, Marco Rossi, Bruno Martino, Mario Tiribelli, Carmen Fava, Arbana Dizdari, Monica Crugnola, Andrea Duminuco, Amedeo Votto, Selene Guerzoni, Giovanni Caocci, Fabrizio Pane, Francesco Lanza, Filippo Branzanti, Alessandra Dedola, Flavia Salvatore, Florian H. Heidel, Massimo Breccia, Giuseppe A. Palumbo, Andrew T. Kuykendall, Alessandro Lucchesi, Sara Galimberti

**Affiliations:** ^1^ Istituto di Ematologia “Seràgnoli” IRCCS Azienda Ospedaliero–Universitaria di Bologna Bologna Italy; ^2^ Hematology and Transplant Unit Azienda Ospedaliera SS Antonio e Biagio e Cesare Arrigo Alessandria Italy; ^3^ Divisione di Ematologia e Unità Trapianto di Midollo Fondazione IRCCS San Gerardo dei Tintori Monza Italy; ^4^ Hematology and Stem Cell Transplant Center AORMN Hospital Pesaro Italy; ^5^ Hematology and Clinical Immunology, Department of Medicine University of Padua Padua Italy; ^6^ Dipartimento di Scienze Mediche e Chirurgiche Università di Bologna Bologna Italy; ^7^ Hematology Section, Department of Clinical Medicine and Surgery University of Naples “Federico II” Naples Italy; ^8^ Hematology Hospital “Andrea Tortora” Pagani Italy; ^9^ Hematology Unit, Department of Clinical and Molecular Sciences, DISCLIMO Università Politecnica delle Marche Ancona Italy; ^10^ Divisione Semplice di Ematologia ASL TO4 Cirié Hospital Turin Italy; ^11^ Azienda Ospedaliera–Universitaria San Luigi Gonzaga Regione Gonzole Orbassano Italy; ^12^ Division of Onco‐Hematology ASST Santi Paolo e Carlo Milan Italy; ^13^ Candiolo Cancer Institute FPO‐IRCCS Candiolo Italy; ^14^ Department of Hematology Polo Universitario Pontino S. M. Goretti Hospital Latina Italy; ^15^ Unit of Hematology, Department of Oncology University of Torino Turin Italy; ^16^ Hematology, Department of Medical Sciences and Public Health University of Cagliari Cagliari Italy; ^17^ University Hematology Division Città della Salute e della Scienza Hospital Turin Italy; ^18^ Division of Hematology Ospedale S. Eugenio Rome Italy; ^19^ Hematology Unit University Hospital “Paolo Giaccone” Palermo Italy; ^20^ Hematology Division Foundation IRCCS Ca' Granda Ospedale Maggiore Policlinico Milan Italy; ^21^ Onco‐Hematology Unit Veneto Institute of Oncology Istituto Oncologico Veneto–IRCCS Padua Italy; ^22^ Department of Hematology‐Oncology Azienda Ospedaliera–Universitaria Renato Dulbecco Catanzaro Italy; ^23^ Division of Hematology Azienda Ospedaliera “Bianchi Melacrino Morelli” Reggio Calabria Italy; ^24^ Division of Hematology and BMT, Department of Medicine University of Udine Udine Italy; ^25^ Department of Clinical and Biological Sciences Azienda Ospedaliera Ordine Mauriziano di Torino University of Turin Turin Italy; ^26^ Division of Hematology, Onco‐Hematologic Department AUSL della Romagna Ravenna Italy; ^27^ Haematology and BMT Centre Azienda Ospedaliero–Universitaria di Parma Parma Italy; ^28^ UO Ematologia AOU Policlinico “G. Rodolico”–San Marco Catania Italy; ^29^ Dipartimento di Medicina Clinica e Sperimentale, UO Ematologia Università di Pisa and AOUP Pisa Italy; ^30^ Hematology Unit IRCCS Istituto Romagnolo per lo Studio dei Tumori “Dino Amadori” Meldola Italy; ^31^ Hematology, Hemostasis, Oncology and Stem Cell Transplantation Hannover Medical School Hannover Germany; ^32^ Division of Cellular Biotechnologies and Hematology Sapienza University Rome Italy; ^33^ Dipartimento di Scienze Mediche, Chirurgiche e Tecnologie Avanzate “G. F. Ingrassia” University of Catania Catania Italy; ^34^ Department of Malignant Hematology H. Lee Moffitt Cancer Center Tampa Florida USA

**Keywords:** anemia, cytopenia, momelotinib, myelofibrosis, thrombocytopenia

## Abstract

**Background:**

Momelotinib, a *JAK1*/*JAK2*/*ACVR1* inhibitor, is approved for treating myelofibrosis with splenomegaly, symptoms, and moderate‐to‐severe anemia. Evidence on its real‐world effectiveness after ruxolitinib failure is limited.

**Methods:**

This study retrospectively analyzed 221 patients who received momelotinib after ruxolitinib failure by assessing spleen, symptom, and anemia responses, safety, and survival. Logistic regression analyses identified the responses’ predictors.

**Results:**

Before momelotinib initiation, all patients had received ruxolitinib for a median of 31.5 months and discontinued as a result of resistance (29.9%), intolerance (48.0%), or both (22.1%). At baseline, all patients started at full dose; 97.3% presented with cytopenia, 34.8% presented with large splenomegaly, and 43.9% were highly symptomatic. Most patients (74.7%) switched from ruxolitinib stop to momelotinib within 2 months without tapering, whereas 18.1% waited over a year. After a median exposure of 8.2 months, adverse events occurred in 35.7% of patients, which prompted dose reductions or permanent discontinuation in 12.7% and 19.9% of cases, respectively. At 6 months, 30.0% achieved ≥50% spleen length reduction (SR50), with higher responses in those with prior SR50 to ruxolitinib and shorter transition intervals. Symptom and anemia responses occurred in 39.2% and 63.4% of cases, respectively. After a median follow‐up of 10.3 months, 11 patients (5.0%) progressed to blast phase, and 37 patients (16.7%) died. Two‐year overall and progression‐free survival (including death and blast phase transformation) were 60.9% and 59.0%, respectively.

**Conclusions:**

Momelotinib demonstrated meaningful clinical benefit and acceptable safety in cytopenic patients pretreated with ruxolitinib, which supports its role after ruxolitinib failure.

## Graphical abstract

Momelotinib demonstrated robust real‐world activity after ruxolitinib failure, with clinically meaningful improvements in spleen (≥50% spleen length reduction, 30.0%), symptoms (≥50% total symptoms score reduction, 39.2%), and anemia (major response, 35.7%) at 6 months. Among patients requiring transfusions at baseline, 37% achieved transfusion independence by 6 months, with overall stable hematologic parameters. Treatment was well tolerated, with adverse events in 35.7% and permanent discontinuation in only 19.9%, which supports sustained dosing in routine clinical practice.

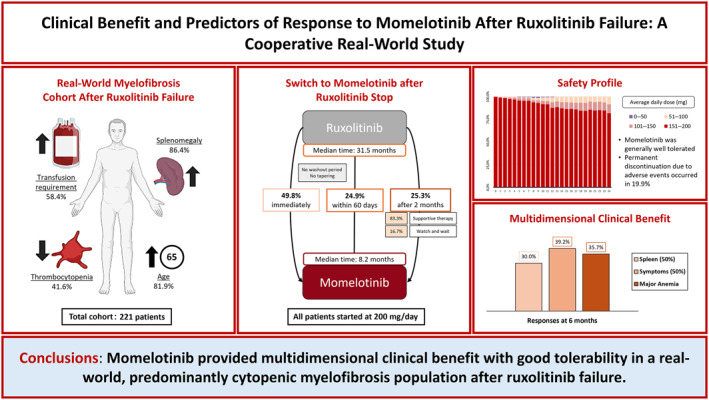

## INTRODUCTION

Myelofibrosis (MF) is a chronic myeloproliferative neoplasm characterized by bone marrow fibrosis, splenomegaly, cytopenias, and a spectrum of constitutional symptoms that significantly impair the quality of life.^1^ Prognosis depends heavily on risk factors such as age, genetics, and disease stage, although significant improvements have been achieved in the last decade as a result of progress in supportive care, disease awareness, and novel agent introduction.^2^ The advent of Janus kinase inhibitors (JAKis), in particular ruxolitinib, has revolutionized MF management by providing effective palliation of splenomegaly and symptom burden and possibly improving survival in selected populations.^3^, ^4^, ^5^ However, resistance/intolerance to ruxolitinib remains a significant challenge, with a substantial proportion of patients ultimately requiring alternative therapies.^6^, ^7^, ^8^, ^9^, ^10^

Momelotinib, a first‐in‐class novel inhibitor targeting *JAK1*, *JAK2*, and activin A receptor type 1 (*ACVR1*), has recently been approved for the treatment of MF presenting with splenomegaly, symptoms, and moderate‐to‐severe anemia.^11^ Unlike ruxolitinib and fedratinib, momelotinib has demonstrated the unique ability to ameliorate anemia, a critical unmet need in MF management.^12^, ^13^, ^14^ Despite promising results from clinical trials, real‐world data on the efficacy and predictors of response to momelotinib, especially as second‐line therapy after ruxolitinib failure, remain limited.^15^, ^16^, ^17^, ^18^

Previous studies have suggested that baseline characteristics, such as spleen size, the presence of cytopenia, peripheral blast count, delayed therapy start, and disease risk, may influence the response to ruxolitinib.^19^, ^20^, ^21^ However, these and other predictors have not been evaluated in a real‐world setting for patients receiving momelotinib.

In this independent cooperative study, we conducted a retrospective analysis of 221 patients with MF who received momelotinib after ruxolitinib failure via a managed access program or according to routine practice. Our objectives were to evaluate spleen, symptom, and anemia responses, as well as to identify baseline and treatment‐related predictors of response to momelotinib. In addition, we comprehensively evaluated key clinical outcomes, including overall survival (OS), progression‐free survival (PFS), and treatment‐related toxicities. These findings aim to inform clinical decision‐making in a challenging, heavily pretreated MF population.

## MATERIALS AND METHODS

### Study design and patient population

This was a multicenter, retrospective, real‐world analysis of 221 patients with a confirmed MF diagnosis receiving momelotinib after ruxolitinib discontinuation.

All patients started momelotinib outside of clinical trials between January 2024 and September 2025.

Patients were treated via a managed access program at 16 Italian participating centers of the RUX‐MF observational study^22^ (ClinicalTrials.gov identifier NCT06516406) (*n* = 125) and in an additional 12 Italian hematology centers (*n* = 64); 32 patients received commercial momelotinib at the H. Lee Moffitt Cancer Center (Tampa, Florida).

### Ethics approval statement

The RUX‐MF study (NCT06516406) was performed in accordance with the guidelines of the institutional review boards of the participating centers and the standards of the Declaration of Helsinki. The promoter of this study was IRCCS Azienda Ospedaliero–Universitaria S. Orsola–Malpighi, Bologna, Italy, which obtained approval from the Area Vasta Emilia Centro Ethics Committee (approval 048/2022/Oss/AOUBo). The study was approved by the local ethics committee of the participating centers (protocol RUX‐MF), and has no commercial support. Informed consent was obtained from all patients involved in the study.

### Data collection

Demographic, clinical, and laboratory data were extracted from electronic medical records, and included age, sex, MF subtype, baseline spleen size (in centimeters below the left costal margin [BLCM]), hemoglobin (Hb) level, platelet (PLT) count, red blood cell (RBC) transfusion requirement, and cytopenic phenotype (Hb < 10 g/dL and/or PLT < 100 × 10^9^/L).^20^ Data on prior ruxolitinib and momelotinib treatment (dose, duration, response, hematologic toxicity, reason for discontinuation, progression to blast phase [BP], and death) were collected. All patients were followed until death or data cutoff (November 2025).

### Definitions

Diagnosis of primary MF and secondary postessential thrombocythemia or polycythemia vera MF was made according to the World Health Organization (WHO) 2022 classification.^23^ Bone marrow biopsies from patients diagnosed before 2022 were reviewed to align with the current criteria. Risk category was assessed at momelotinib start according to the Dynamic International Prognostic Scoring System.^24^ Evolution to BP was defined by leukemic blast cells being at least 20% in peripheral blood or bone marrow according to WHO criteria.^23^

High molecular risk (HMR) pathogenetic mutations were defined as those including *ASXL1*, *SRSF2*, *EZH2*, *IDH1*, *IDH2*, and *U2AF1*.^25^

Responses were assessed at 3 and 6 months after initiation of momelotinib.

Given the real‐life setting of the study, we adopted a dual approach to ensure a comprehensive assessment of spleen response (SR). First, SR was assessed according to International Working Group–Myeloproliferative Neoplasms Research and Treatment (IWG‐MRT) criteria, which require a baseline palpable spleen of ≥5 cm BLCM.^22^ In parallel, we measured the relative reduction in spleen size, defined as a decrease in palpable spleen length by at least 30% (SR30) or 50% (SR50) from baseline in all patients with measurable splenomegaly. This allowed us to capture clinically meaningful changes even in patients with less pronounced splenomegaly who would not have met the stringent IWG‐MRT threshold.

Symptom response (SyR) was defined as a reduction in total symptom score (TSS)^26^ by at least 30% (SyR30) or 50% (SyR50) from baseline, and was assessed in patients with a baseline TSS of greater than 0. SyR50 corresponds to IWG‐MRT criteria.^27^

Anemia response (AR) was defined according to updated European LeukemiaNet (ELN) criteria.^28^ Briefly, a major response was defined as the absence of transfusions in the preceding 3 months for transfusion‐dependent (TD) patients, and as an increase in Hb of greater than 1.5 g/dL from baseline for non‐TD patients. A minor response required a ≥50% reduction in TD patients or an Hb increase exceeding 1.0 g/dL in non‐TD patients.

### Statistical analysis

Statistical analysis was performed at the biostatistics laboratory of the Myeloproliferative Neoplasms Unit at the Institute of Hematology “L. and A. Seràgnoli,” IRCCS Azienda Ospedaliero Universitaria di Bologna. Descriptive statistics were used to summarize baseline characteristics and response rates. Continuous variables were expressed as mean ± standard deviation (SD) or median and range, as appropriate. Categorical variables were reported as frequencies and percentages.

Univariate/multivariate logistic regression analyses were conducted to identify predictors of IWG‐MRT spleen, symptom, and updated ELN anemia responses at 3 and/or 6 months.

Covariates were selected on the basis of clinical experience and prior studies, which aimed to identify key factors that may influence treatment outcomes.^19^, ^24^, ^29^, ^30^ These included demographic and clinical characteristics, as well as treatment‐related variables: older age (>65 years), sex, MF subtype, type of driver mutation (CALR type 1 mutated vs. other mutations), higher risk category, large splenomegaly (palpable spleen length > 10 cm BLCM), baseline PLT count of >200 × 10^9^/L, high peripheral blast count (≥3%), RBC transfusion requirement, high symptom burden (TSS, ≥20), short transition time between ruxolitinib and momelotinib (<2 months), shorter duration of ruxolitinib therapy (<2 years), momelotinib use in second‐line (vs. third‐line) treatment, and prior spleen and symptom response to ruxolitinib. Odds ratios (ORs) with 95% confidence intervals (CIs) were reported. A two‐sided *p* value of <.05 was considered statistically significant.

OS and PFS were calculated with Kaplan–Meier curves from the date of momelotinib start to the date of death or last contact (OS) or to the date of BP, death, or last contact (PFS), whichever came first. Patients who underwent allogeneic stem cell transplantation were censored at the date of the procedure. The median time to the first adverse event was also estimated with the Kaplan–Meier method.

The log‐rank test was applied to compare PFS between patients who showed combined improvement in splenomegaly and anemia and those who did not.

All analyses were performed with STATA/SE software, version 18.5 (StataCorp).

## RESULTS

### Patient characteristics at momelotinib initiation

A total of 221 patients were included in this analysis. Baseline characteristics reflected an advanced‐disease stage, and are summarized in Table 1. All patients were in chronic phase at momelotinib start.

**TABLE 1:** Baseline characteristics of the study cohort (*N* = 221).

Characteristic	
Age, years	
Mean (±SD)	72.2 (8.4)
>65, No. (%)	181 (81.9)
>75, No. (%)	96 (43.4)
Male sex, No. (%)	126 (57.0)
MF, No. (%)	
Primary	130 (58.8)
Post‐PV	40 (18.1)
Post‐ET	51 (23.1)
Hemoglobin, g/dL	
Mean (±SD)	8.8 (1.2)
<10, No. (%)	207 (93.7)
<8, No. (%)	130 (58.8)
RBC transfusion requirement, No. (%)	129 (58.4)
RBC transfusion dependence,^a^ No. (%)	92 (41.6)
Platelets, ×10^9^/L	
Mean (±SD)	172.8 (160.4)
<200, No. (%)	155 (70.1)
<100, No. (%)	92 (41.6)
<50, No. (%)	29 (13.1)
Cytopenic phenotype, No. (%)	215 (97.3)
Peripheral blast count	
Mean (±SD), %	1.1 (1.6)
≥3%, No. (%)	32 (14.5)
≥1%, No. (%)	95 (43.0)
Palpable spleen, No. (%)	191 (86.4)
Spleen length, cm BLCM	
Mean (±SD)	7.8 (6.5)
≥5, No. (%)	145 (65.6)
≥10, No. (%)	77 (34.8)
TSS	
Mean (±SD)	20.0 (15.3)
≥1, No. (%)	194 (87.8)
≥20, No. (%)	97 (43.9)
Driver mutations, No. (%)	
*JAK2*	138 (62.4)
*CALR*	54 (24.4)
*MPL*	25 (11.3)
Triple‐negative	4 (1.8)
*CALR* mutations, No. (%)	
Type 1/type 1–like	24 (10.9)
Non–type 1 or unknown	30 (13.6)
Wild‐type	167 (75.6)
High molecular risk mutations, No. (%)	29 of 66 (43.9)
*ASXL1*	20 (30.3)
*EZH2*	3 (4.5)
*SRSF2*	6 (9.1)
*IDH1/2*	3 (4.5)
*U2AF1*	5 (7.6)
DIPSS risk score, No. (%)	
Intermediate 1	24 (10.9)
Intermediate 2	141 (63.8)
High	56 (25.3)

Abbreviations: BLCM, below the left costal margin; DIPSS, Dynamic International Prognostic Scoring System; ET, essential thrombocythemia; MF, myelofibrosis; PV, polycythemia vera; RBC, red blood cell; SD, standard deviation; TSS, total symptom score.

^a^ Transfusion dependence was defined as ≥3 units in the 12 weeks before baseline.^24^

A cytopenic phenotype was observed in 215 patients (97.3%), with 58.4% of patients requiring RBC transfusions and 41.6% having a PLT count of <100 × 10^9^/L. In 29 patients (13.1%), the PLT count was less than 50 × 10^9^/L.

Spleen was palpable in 86.4% of patients; 65.6% had a spleen length of ≥5 cm, and 34.8% had a spleen length of ≥10 cm BLCM. TSS was equal or higher than 20 in 43.9% of patients.

HMR mutations were detected in 29 patients (of 66 available; 43.9%).

### Transition from ruxolitinib to momelotinib

The median duration of prior ruxolitinib therapy was 31.5 months (range, 0.3–114.6 months). The median ruxolitinib dose at the time of treatment discontinuation was 10 mg twice daily (bid). Specifically, 42.5% of patients were receiving a lower dose than that recommended by the prescribing indication on the basis of PLT count. The distribution of doses at discontinuation was 5 mg bid (46.1%; *n* = 102), 10 mg bid (29.9%; *n* = 66), 15 mg bid (12.2%; *n* = 27), and 20 mg bid (11.8%; *n* = 26).

Reasons for ruxolitinib discontinuation included resistance in 29.9% (lack/loss of spleen and/or symptom response), adverse events in 48.0%, or both resistance and intolerance in 22.1%. Most adverse events leading to ruxolitinib discontinuation were anemia and/or thrombocytopenia ( 67.4%; *n* = 149).

In most cases, the transition from ruxolitinib to momelotinib was rapid, without intermediate alternative treatment: 49.8% of patients switched immediately (no washout period), and 24.9% initiated within 2 months from ruxolitinib stop. Ruxolitinib tapering was never performed. The remaining 56 patients (25.3%) started momelotinib after a median of 18 months (range, 2.1–30.8 months); overall, in 18.1% of patients, latency to momelotinib treatment was longer than 1 year (Figure S1). Among these patients, 22 received second‐line fedratinib, 25 received supportive therapy—including RBC transfusions, danazol, erythropoietin‐stimulating agents, and hydroxyurea—whereas the remaining nine patients (16.1%) were managed with a watch‐and‐wait approach. Among these nine patients, six had PLT counts of <50 × 10^9^/L, one had an Hb level of <8 g/dL, and two had recently discontinued ruxolitinib because of grade 3 infections.

Notably, one patient (dose at ruxolitinib stop, 15 mg bid) experienced ruxolitinib discontinuation syndrome after direct transition to momelotinib. Five days after switching, the patient developed severe fatigue (score, 9 of 10), accompanied by dyspnea and significant oxygen desaturation. Infectious, cardiac, and pulmonary causes were ruled out. The patient's condition improved with the administration of oral corticosteroids.

### Momelotinib safety

**FIGURE 1:**
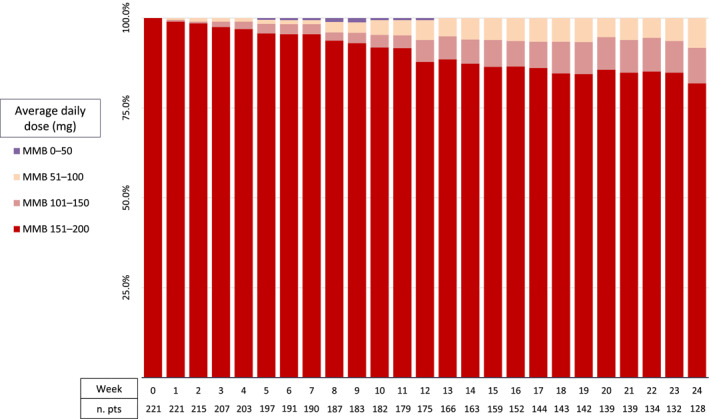
Momelotinib dose intensity in the overall cohort across the first 24 weeks. MMB indicates momelotinib; n. pts, number of patients.

The median duration of momelotinib therapy was 8.2 months (range, 0.2–49.9 months). All patients began at the full approved dose of 200 mg once daily, and 82.4% maintained the full dose throughout the first 24 weeks of therapy (Figure 1). Overall, 79 patients (35.7%) experienced a total of 104 momelotinib‐related adverse events (Table 2). The median time from initiation of momelotinib therapy to the first adverse event was 13.1 months (95% CI, 11.6 months to not reached).

**TABLE 2:** Adverse events during momelotinib therapy.

Category	Type	Grade	No. (%)	Patients, No.; action required
Gastrointestinal (*n* = 16)	Diarrhea	2	6 (2.7)	4; temporary dose reduction, then restored at full dose 2; loperamide administration
		3	3 (1.4)	3; permanent discontinuation
	Nausea	1	2 (0.9)	2; monitoring
		2	2 (0.9)	1; antiemetic therapy administration 1; antiemetic therapy administration and stable dose reduction
	Vomiting	2	2 (0.9)	1; permanent discontinuation 1; antiemetic therapy administration
	Hyperamylasemia	2	1 (0.5)	1; stable dose decrease
Hepatic (*n* = 4)	Transaminitis	2	4 (1.8)	1; monitoring 2; stable dose reduction 1; permanent discontinuation
Hematologic (*n* = 31)	Anemia	2	3 (1.4)	1; red blood cell transfusion support 1; stable dose reduction 1; permanent discontinuation
		3	3 (1.4)	3; permanent discontinuation
	Thrombocytopenia	2	6 (2.7)	1; temporary dose reduction, then restored at full dose 5; stable dose reduction
		3	9 (4.1)	2; stable dose reduction 1; danazol administration 6; permanent discontinuation
		4	10 (4.5)	2; stable dose reduction 8; permanent discontinuation
Renal (*n* = 8)	Macrohematuria	3	1 (0.5)	1; monitoring
	Creatinine increase	2	1 (0.5)	1; monitoring
		3	1 (0.5)	1; permanent discontinuation
	Renal failure	3	5 (2.3)	1; stable dose reduction 4; permanent discontinuation
Neurological (*n* = 12)	Somnolence	1	1 (0.5)	1; stable dose reduction
	Migraine	2	2 (0.9)	1; temporary dose reduction, then restored at full dose 1; stable dose reduction
		3	2 (0.5)	1; temporary interruption, then restarted at full dose 1; permanent discontinuation
	Peripheral neuropathy	1	2 (0.9)	1; monitoring 1; stable dose reduction
		2	2 (0.9)	2; stable dose reduction
		3	1 (0.5)	1; permanent discontinuation
	Visual hallucinations	3	1 (0.5)	1; permanent discontinuation
	Epilepsy	3	1 (0.5)	1; permanent discontinuation
Infectious (*n* = 15)	Urinary tract infection	2	2 (0.9)	2; antibiotic therapy administration
		3	4 (1.8)	2; temporary interruption, then restarted at a lower dose 2; permanent discontinuation
	Bronchitis	2	2 (0.9)	2; antibiotic administration
	Pneumonia	3	2 (0.9)	1; antibiotic therapy administration 1; permanent discontinuation
	Septic shock	3	1 (0.5)	1; permanent discontinuation
		4	4 (1.8)	4; permanent discontinuation
Dermatological (*n* = 5)	Cutaneous rash	2	3 (1.4)	2; topical therapy administration 1; temporary interruption, then restarted at a lower dose
	Squamous cell carcinoma	3	2 (0.9)	1; surgical excision 1; permanent discontinuation
Cardiovascular (*n* = 2)	Hypotension	2	1 (0.5)	1; monitoring
	Pericarditis	3	1 (0.5)	1; antibiotic therapy administration and permanent discontinuation
Miscellaneous (*n* = 11)	Muscle cramps	1	3 (1.4)	3; monitoring
	Fatigue	2	1 (0.5)	1; monitoring
		3	1 (0.5)	1; permanent discontinuation
	Lack of appetite	1	2 (0.9)	1; temporary interruption, then restarted at a lower dose 1; permanent discontinuation
	Weight loss	1	2 (0.9)	2; monitoring
	Rhinorrhea	1	1 (0.5)	1; stable dose reduction
	Dysuria	3	1 (0.5)	1; permanent discontinuation

Gastrointestinal toxicities were the most frequent nonhematologic events (*n* = 16; 7.2%), predominantly diarrhea (*n* = 9; 4.1%). Grade 2 gastrointestinal events were generally manageable with supportive care/dose reduction, whereas all grade 3 adverse events (*n* = 3) led to treatment discontinuation. Infectious events accounted for 15 cases (6.8%), of which 11 (5.0%) were grade 3 (*n* = 4, urinary tract; *n* = 2, pneumonia; *n* = 5, septic shock) and eight (3.6%) caused discontinuation. Grade 3 neurological adverse events occurred in five patients (2.3%; namely migraine, peripheral neuropathy, visual hallucinations, and epilepsy, all resulting in treatment discontinuation); four additional cases of low‐grade peripheral neuropathy were documented. Overall, six patients (2.7%) had severe renal toxicity (grade 3 creatinine increase or renal failure). Two cases of cutaneous squamous cell carcinoma were observed, one in a patient with long‐standing hydroxyurea exposure.

Globally, adverse events required transient/stable dose reductions in 28 patients (12.7%), and prompted momelotinib permanent discontinuation in 44 patients (19.9%). The most common adverse event leading to momelotinib discontinuation was thrombocytopenia (*n* = 14; 6.3% discontinuation rate). Of these, seven patients started momelotinib with a PLT count of <50 × 10^9^/L, and four patients started momelotinib with a PLT count between 50 and 100 × 10^9^/L.

During the first 6 months of therapy, PLT counts remained stable, with a mean of 172.8 (±SD 160.4) at baseline, 179.7 (±SD 169.2) at 3 months, and 168.0 (±SD 167.0) at 6 months, which showed no significant reductions.

At momelotinib start, 78 of 207 patients with anemia (37.7%) were under therapy with antianemia drugs (erythropoietin‐stimulating agents, 34.8%; danazol, 2.9%); three patients received iron chelators. The combination therapy was safe and well tolerated.

Overall, 68 patients (30.8%) discontinued momelotinib during follow‐up. Reasons for discontinuation included resistance in 16 patients (7.2% of the overall cohort; 23.5% among those who discontinued), adverse events in 19 patients (8.6%; 27.9%), combined resistance and intolerance in 25 patients (11.3%; 36.8%), progression to BP in five patients (2.3%; 7.4%), and allogeneic stem cell transplantation in three responding patients (1.4%; 4.4%).

### Response rates and predictors

At baseline, 191 patients had a palpable spleen; 143 and 100 were evaluable for SR at 3 and 6 months, respectively.

At 3 months, SR30 and SR50 were achieved by 35.7% and 23.8% of patients, respectively. These rates improved to 45.0% and 30.0% at 6 months. By applying IWG‐MRT criteria (108 and 77 evaluable patients at 3 and 6 months, respectively), response rates were 13.0% and 16.9% at 3 and 6 months, respectively.

**FIGURE 2:**
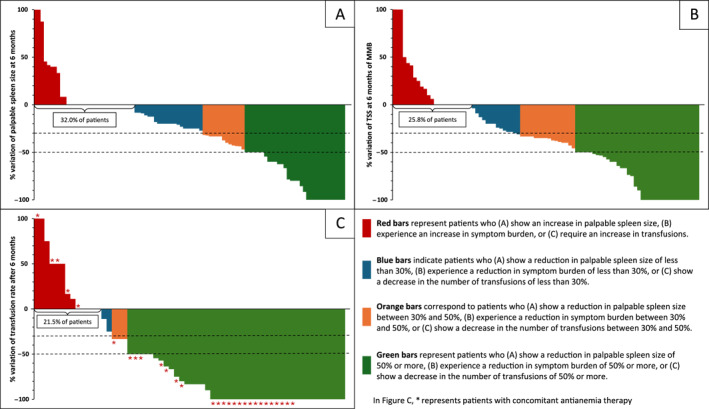
Variations in palpable spleen (A), total symptom score (B), and transfusion rate (C) at 6 months. MMB indicates momelotinib; TSS, total symptom score.

The proportion of patients with stable/increasing splenomegaly decreased from 40.6% (3 months) to 32.0% (6 months) (Figure S2A; Figure 2A).

In multivariate logistic regression, achieving an SR50 at 6 months during prior ruxolitinib (OR, 6.25; 95% CI, 1.02–10.25; *p* = .048) and a transition time between ruxolitinib and momelotinib of <2 months (OR, 2.65; 95% CI, 1.04–8.40; *p* = .045) remained independent predictors of a higher probability of achieving an IWG‐MRT SR to momelotinib at 3 and/or 6 months (Figure S3).

At baseline, 194 patients reported MF‐associated symptoms (TSS, ≥1); 144 and 97 patients were evaluable for symptom response at 3 and 6 months, respectively.

SyR30 and SyR50 were achieved by 45.8% and 23.7% of patients at 3 months, and increased to 59.8% and 39.2% at 6 months, respectively.

Between 3 and 6 months, the proportion of nonresponsive patients declined from 33.6% to 25.3%, which suggested progressive symptom relief over time. Patients with stable or worsening symptoms were mostly elderly individuals (aged >65 years, 88%; >70 years, 70%; >75 years, 39%), with a higher baseline TSS (mean, 23.9 vs. 16.4; *p* < .001). None of the asymptomatic patients at baseline developed new symptoms (Figure S2B; Figure 2B).

**FIGURE 3:**
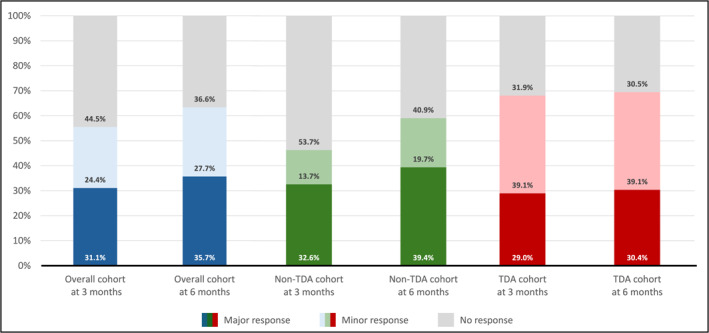
Anemia response at 3 and 6 months in transfusion‐dependent and non–transfusion‐dependent patients. TDA indicates transfusion‐dependent anemia.

At 3 months, 55.5% of patients achieved an AR (major, 31.1%). The proportion of responders increased at 6 months (63.4%; major, 35.7%) (Figure 3). In the TD cohort, the median frequency of RBC transfusions decreased from 6 to 2.5 units at 3 months and to 2 units at 6 months. In the non‐TD group, the mean Hb level at baseline was 9.3 g/dL (±SD 1.1), increased to 10.3 g/dL (±SD 1.5) at 3 months, and then remained stable at 6 months (10.4 g/dL ± SD 1.6). Globally, 37.0% of evaluable patients at 6 months who required transfusions at baseline achieved complete transfusion independence between months 3 and 6. Notably, these patients exhibited a numerically more pronounced mean percentage reduction compared with those who did not achieve complete transfusion independence (−39.6% ± SD 34.1 vs. −24.6% ± SD 51.2; *p* = .21).

The proportion of patients with stable/increasing transfusion need was stable between 3 (22.0%) and 6 months (21.5%) (Figure S2C; Figure 2C).

A short transition time (<2 months) between ruxolitinib and momelotinib (OR, 2.04; 95% CI, 1.03–4.00; *p* = .043) was an independent predictor of a higher probability of achieving an AR at 3 and/or 6 months.

Combined IWG‐MRT response rates at 3 months were as follows: SR + SyR, 1.1%; SR + AR, 6.3%; SyR + AR, 8.4%; and triple response, 1.1%. At 6 months, these proportions increased, primarily driven by a significant improvement in symptom relief (SR + SyR, 8.8%; SR + AR, 13.2%; SyR + AR, 25.0%; triple response, 5.9%).

### Survival outcomes

After a median follow‐up of 10.3 months (range, 0.2–51.3 months), 11 patients (5.0%) progressed to BP, 17 (7.7%) died on momelotinib, and 20 (9.0%) died after discontinuation. Among the 60 patients who discontinued momelotinib during chronic phase, subsequent therapies included fedratinib in six patients (10.0%), hydroxyurea in four (6.7%), pacritinib in three (5.0%), protocol‐based treatments in six (10.0%), ruxolitinib rechallenge in 18 (30.0%), and supportive care in 23 (38.3%; six, only blood transfusions; eight, erythropoietin‐stimulating agents; four, danazol; two, splenic radiotherapy; three, corticosteroids).

**FIGURE 4:**
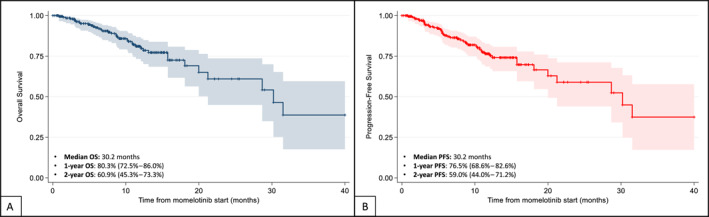
Overall survival (A) and progression‐free survival (B) Kaplan–Meier curves. OS indicates overall survival; PFS, progression‐free survival (including death and progression to blast phase).

The estimated median OS was 30.2 months, with a 2‐year OS of 60.9% (95% CI, 45.3%–73.3%) (Figure 4A). The median PFS was 30.2 months, with a 2‐year PFS of 59.0% (95% CI, 44.0%–71.2%) (Figure 4B).

A significant correlation was observed between the early achievement of combined spleen reduction plus hemoglobin increase and longer PFS. Patients achieving both improvements at 3 months had a 2‐year PFS of 76.1% (95% CI, 53.0%–88.9%) versus 42.6% in nonresponders (95% CI, 20.4%–63.3%; *p* = .028) (Figure S4A). Because of improvements at 6 months, this difference was maintained (2‐year PFS, 82.0% vs. 56.9%; *p* = .034) (Figure S4B).

## DISCUSSION

This independent, real‐world study provides insights into the safety, efficacy, and predictors of response to second‐line momelotinib in patients with MF who previously failed ruxolitinib therapy.

In our real‐world cohort, adverse events were generally consistent with those reported in pivotal phase 3 trials (SIMPLIFY‐1/SIMPLIFY‐2/MOMENTUM) and with other real‐life experiences.^12^, ^15^, ^31^, ^32^

Gastrointestinal toxicities—mainly diarrhea—were the most common nonhematologic adverse events, which occurred in 7.2% of patients and were generally manageable with supportive care/dose modifications.

Grade ≥3 infections occurred in 5% of cases, slightly lower than the 7% reported in SIMPLIFY‐1, and led to permanent treatment discontinuation in 3.6% of the cohort.

In an integrated analysis of the three phase 3 trials, grade ≥3 neurological events were reported in 1.2% of patients, comparable to the 0.5% observed in our series. In contrast, earlier and more recent Mayo Clinic reports have documented a higher incidence of predominantly low‐grade peripheral neuropathy, reported in 44% of patients in 2015, and as treatment emergent in 20% of patients in 2026, which underscores the need for careful neurological monitoring over time.^32^, ^33^, ^34^

The same integrated analysis documented 35 nonmelanoma skin cancers (grade 3 in 0.6%).^32^ In our cohort, two grade 3 cases were observed, which highlights the importance of dermatologic surveillance during therapy with JAKis, including momelotinib.

A mild creatinine increase (grade 1/2) was reported in 16.7% of patients in the MoReLife study,^15^ whereas it occurred in only 0.5% of our patients; an additional 2.6% experienced severe renal dysfunction. Minor changes in creatinine may have been underreported; however, multidisciplinary monitoring remains essential for timely identification and management of adverse events.

Despite the advanced‐disease features of this cohort, more than 30% of patients achieved a ≥50% reduction in spleen size after transitioning to momelotinib. This evidence matches previous real‐world reports, including the Spanish GEMFIN (24.4%)^16^ and German MoReLife (25%)^15^ studies, which also included patients treated in the front‐line setting.

Notably, all IWG‐MRT SRs were evaluated as a reduction of spleen length in patients with baseline palpable splenomegaly, and are therefore attributable to momelotinib rather than residual ruxolitinib effects. Among patients classified as nonresponders, however, a large proportion (94%) with prior SR to ruxolitinib showed no reexpansion of splenomegaly. This suggests that momelotinib, although not inducing a formal response by IWG‐MRT criteria in these cases, effectively maintained the clinical benefit achieved with ruxolitinib by preventing spleen enlargement over time.

Symptom improvement was also observed, with nearly 60% achieving a ≥30% reduction in symptom burden at 6 months. These results are consistent with results from pivotal clinical trials of momelotinib.^12^, ^35^

A central finding of our analysis is the predictive value of previous SR to ruxolitinib. Achieving SR50 during the first 6 months of ruxolitinib treatment was strongly correlated with SR to momelotinib. This supports the hypothesis that an early response to one JAKi would reflect an underlying disease biology intrinsically more sensitive to JAK‐STAT pathway suppression, rather than responsiveness to a specific agent.^36^

Another clinically relevant predictor that emerged from our multivariate analysis is the transition interval between ruxolitinib and momelotinib. Patients who switched within 2 months were significantly more likely to achieve both spleen and anemia responses. The close temporal switch from ruxolitinib to momelotinib in most patients suggests that part of the early (3‐month) hemoglobin rise may stem from the removal of ruxolitinib‐related suppression rather than from immediate momelotinib activity. However, the sustained improvement observed by 6 months strongly aligns with momelotinib *ACVR1*‐driven hepcidin suppression, which indicates a substantive and durable pharmacologic effect. Notably, longer transition intervals likely reflect more advanced disease or clinical instability, whereas rapid switching may favorably influence outcomes by enabling earlier initiation of momelotinib before further decline of marrow reserve.

This observation is consistent with SIMPLIFY‐1 subanalysis, where immediate switching resulted in rapid Hb gains of approximately 1 g/dL within the first month.^37^

Overall, these data support the timely sequencing of JAKis, and caution against unnecessary washout periods during transition. Additionally, we report, for the first time, a possible case of ruxolitinib discontinuation syndrome^38^ in a patient who underwent a direct switch to momelotinib. Although preliminary and requiring validation, this observation underscores the need for caution and strict follow‐up when managing such transitions.

Importantly, baseline factors traditionally associated with poor outcomes—older age, severe cytopenias, transfusion dependence, and large spleen—did not diminish the likelihood of response. This is clinically relevant, given that cytopenias often restrict the use of other JAKis and are a major challenge after ruxolitinib failure.^20^, ^39^, ^40^ The preserved efficacy in cytopenic patients likely reflects a momelotinib distinctive mechanism of action by combining *JAK1/2* inhibition with *ACVR1* blockade, and thereby modulating hepcidin and improving iron availability. Consistent with this rationale, our cohort experienced significant improvements in Hb levels and transfusion burden, which reinforces momelotinib‐integrated activity against splenomegaly, symptoms, and anemia.^41^, ^42^

In the SIMPLIFY‐2 and MOMENTUM trials, conducted in ruxolitinib‐experienced patients, momelotinib consistently improved anemia and increased transfusion independence.^43^, ^44^, ^45^ In SIMPLIFY‐2, it outperformed the best available therapy, including concomitant administration of ruxolitinib and antianemia agents, in the reduction of transfusion needs and in the control of splenomegaly and symptoms.^44^ Similarly, in MOMENTUM, it achieved higher rates of transfusion independence and hemoglobin increase compared to danazol.^46^ These prospective results are mirrored in our real‐world experience, where approximately one third of patients achieved a major AR after 6 months. Anemia improvement may also reduce health care resource utilization and patient burden. Data show that TD patients with MF require more hospital time and incur higher costs compared to nonanemic or non‐TD patients, with transfusion‐related care representing a major driver of economic and organizational burden.^47^, ^48^, ^49^ Remarkably, these results are consistent with the Desirability of Myelofibrosis Outcomes framework established by the ELN, which prioritizes AR as a key therapeutic goal in MF management.^50^

Survival outcomes in our cohort were comparable to those documented in clinical trials. The 2‐year OS rate of 60.9% closely matches the 65.8% reported in SIMPLIFY‐2; PFS was similarly aligned.^43^ Notably, early combined improvements in spleen size and hemoglobin^51^ were associated with better PFS, which suggests that a multidimensional response may serve as a meaningful prognostic indicator.

Our study has several limitations. Its retrospective, real‐world design introduces inherent risks of selection and reporting biases, including potential underestimation of adverse events, variability in spleen and symptom response assessments, and heterogeneity in transfusion practices across centers. Specifically, spleen size was assessed exclusively by clinical palpation. This reflects the reality of routine practice in the participating centers, where serial ultrasound examinations are not performed at predefined intervals, and therefore were not available for systematic evaluation. Moreover, molecular and cytogenetic data—key determinants of prognosis and treatment response—were not systematically available, and could not be incorporated into the analysis. Prospective studies integrating comprehensive clinical and biological profiling are therefore needed to validate and extend these findings.

In conclusion, momelotinib provided meaningful benefit in a large, predominantly cytopenic real‐world population. Prior SR to ruxolitinib and a short transition interval were key predictors of efficacy, whereas baseline cytopenias did not compromise treatment outcomes. These findings support momelotinib as a valuable therapeutic option after ruxolitinib failure, and offer practical guidance for treatment sequencing in MF.

## AUTHOR CONTRIBUTIONS

**Francesca Palandri**: Conceptualization; investigation; writing—original draft; funding acquisition; writing—review and editing; visualization; data curation; and resources. **Monia Marchetti**: Conceptualization; investigation; writing—review and editing; visualization; and resources. **Elena M. Elli**: Conceptualization; investigation, visualization; writing—review and editing; and resources. **Alessandro Isidori**: Resources; investigation; and writing—review and editing. **Ilaria Gianesello**: Investigation and resources. **Chiara Sartor**: Investigation; writing—review and editing; and resources. **Novella Pugliese**: Investigation; writing—review and editing; and resources. **Maria Di Perna**: Investigation; writing—review and editing; and resources. **Erika Morsia**: Investigation; writing—review and editing; and resources. **Susanna Gallo**: Investigation; writing—review and editing; and resources. **Federico Itri**: Investigation; writing—review and editing; and resources. **Vittorio Montefusco**: Investigation; writing—review and editing; and resources. **Elena Crisà**: Investigation; writing—review and editing; and resources. **Annalisa Biagi**: Investigation; writing—review and editing; and resources. **Eloise Beggiato**: Investigation; writing—review and editing; and resources. **Olga Mulas**: Investigation; writing—review and editing; and resources. **Giulia Benevolo**: Investigation; writing—review and editing; and resources. **Elisabetta Abruzzese**: Investigation; writing—review and editing; and resources. **Marco Santoro**: Investigation; writing—review and editing; and resources. **Alessandra Iurlo**: Investigation; writing—review and editing; and resources. **Elisabetta Calistri**: Investigation; writing—review and editing; and resources. **Marco Rossi**: Investigation; writing—review and editing; and resources. **Bruno Martino**: Investigation; writing—review and editing; and resources. **Mario Tiribelli**: Investigation; writing—review and editing; and resources. **Carmen Fava**: Investigation; writing—review and editing; and resources. **Arbana Dizdari**: Investigation; writing—review and editing; and resources. **Monica Crugnola**: Investigation; writing—review and editing; and resources. **Andrea Duminuco**: Investigation; writing—review and editing; and resources. **Amedeo Votto**: Investigation; writing—review and editing; and resources. **Selene Guerzoni**: Investigation; writing—review and editing; and resources. **Giovanni Caocci**: Investigation; writing–review and editing; and resources. **Fabrizio Pane**: Investigation; writing—review and editing; and resources. **Francesco Lanza**: Investigation; writing—review and editing; and resources. **Filippo Branzanti**: Conceptualization; writing–original draft; writing—review and editing; visualization; formal analysis; and data curation. **Alessandra Dedola**: Investigation; writing—review and editing; and resources. **Flavia Salvatore**: Investigation; writing—review and editing; and resources. **Florian H. Heidel**: Investigation; writing—review and editing; and resources. **Massimo Breccia**: Investigation; writing—review and editing; and resources. **Giuseppe A. Palumbo**: Investigation; writing–review and editing; and resources. **Andrew T. Kuykendall**: Investigation; writing—review and editing; and resources. **Alessandro Lucchesi**: Conceptualization; investigation; writing—review and editing; resources; and visualization. **Sara Galimberti**: Conceptualization; investigation; visualization; writing—review and editing; and resources.

## CONFLICT OF INTEREST STATEMENT

Francesca Palandri reports receiving honoraria and consulting fees from Novartis, GlaxoSmithKline (GSK), Bristol‐Myers Squibb (BMS), Incyte, Sanofi, Takeda, Sobi, and AOP Health. Elena M. Elli reports serving on meeting/advisory boards for Novartis, GSK, AbbVie, and AOP Health. Elena Crisà reports receiving consulting fees from GSK. Elisabetta Abruzzese reports receiving honoraria and consulting fees from Novartis, GSK, BMS, Incyte, Ascentage, and Pfizer. Marco Rossi reports receiving pharmaceuticals from GSK. Mario Tiribelli reports receiving honoraria from and serving on speakers’ bureaus for Novartis, BMS, Pfizer, and Incyte. Arbana Dizdari reports receiving travel support to the institution from BeOne Medicines, GSK, and Novartis. Monica Crugnola reports receiving honoraria from Novartis and Amgen. Amedeo Votto reports receiving consulting fees and honoraria from GSK. Fabrizio Pane reports receiving honoraria from Incyte, Novartis, Jazz, BMS/Celgene, Amgen, and Gilead. Florian H. Heidel reports receiving consulting fees from BMS/Celgene, Novartis, CTI BioPharma, AOP Orphan Pharmaceuticals, Janssen, GSK, Merck Sharp & Dohme, AbbVie, Takeda, Kartos, Geron, Silence Therapeutics, Prelude, and Sumitomo, and research funding from BMS/Celgene and Novartis. Massimo Breccia reports receiving honoraria from Novartis, BMS, Pfizer, and Incyte. Giuseppe A. Palumbo reports receiving consulting fees and honoraria from AbbVie, AOP Orphan Pharmaceuticals, AstraZeneca, BMS, Incyte, GSK, MorphoSys, and Novartis, and travel support from Janssen, BeiGene, AbbVie, AstraZeneca, Stemline, and Sobi. Andrew T. Kuykendall reports receiving grants, personal fees, and/or nonfinancial support from GSK, AbbVie, Protagonist, Silence Therapeutics, Blueprint Medicines, MorphoSys/Novartis, Incyte, Geron, Janssen, Sobi, Cogent, Kartos, BMS, Sierra Oncology, Constellation, Prelude, CTI BioPharma, Karyopharm, PharmaEssentia, Imago BioSciences, Celgene, and Takeda. Alessandro Lucchesi reports receiving consulting fees from GSK, Novartis, and Stemline. The other authors declare no conflicts of interest.

## Supporting information

Supplementary Material

## Data Availability

The data that support the findings of this study are available from the corresponding author on reasonable request (https://zenodo.org/records/17485011).
