# Supplementary material for: Clinical benefit and predictors of response to momelotinib after ruxolitinib failure: A cooperative real‐world study
Source: Cancer. 2026 May 12;132:e70457. doi: 10.1002/cncr.70457 (PMC13166382; doi:10.1002/cncr.70457)

Supplemental Figure 1: Transitioning From Ruxolitinib to Momelotinib

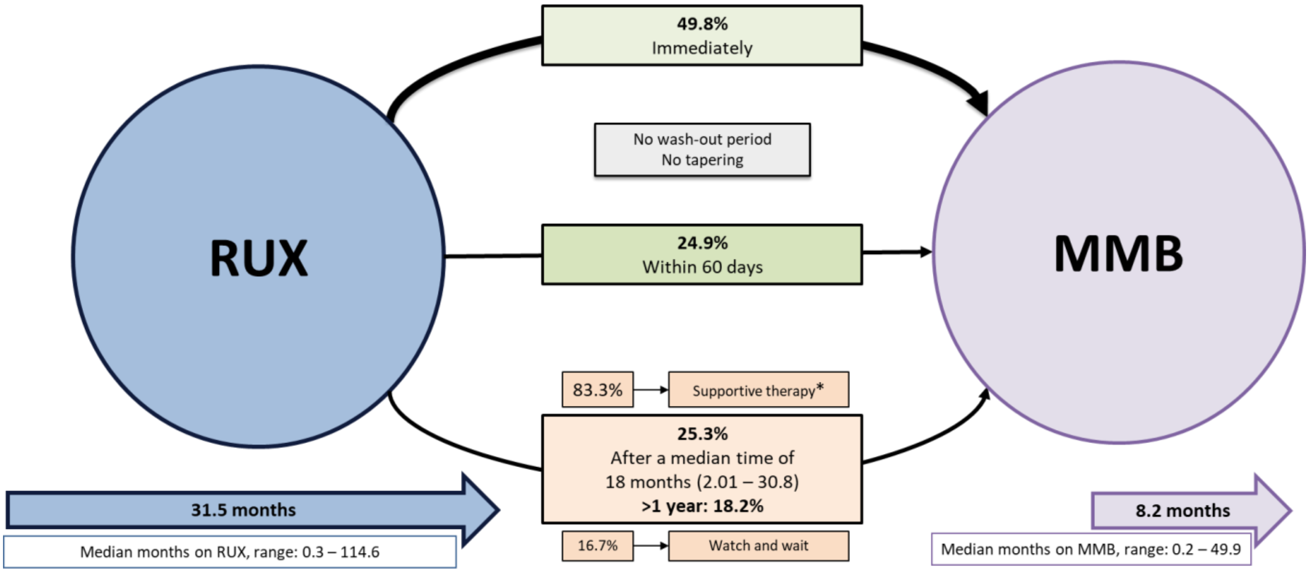

Supplemental Figure 1*: *including 22/56 with Fedratinib*

Supplemental Figure 2: Palpable spleen (a) and total symptoms score (b) and transfusion rate (c) variations at 3 months

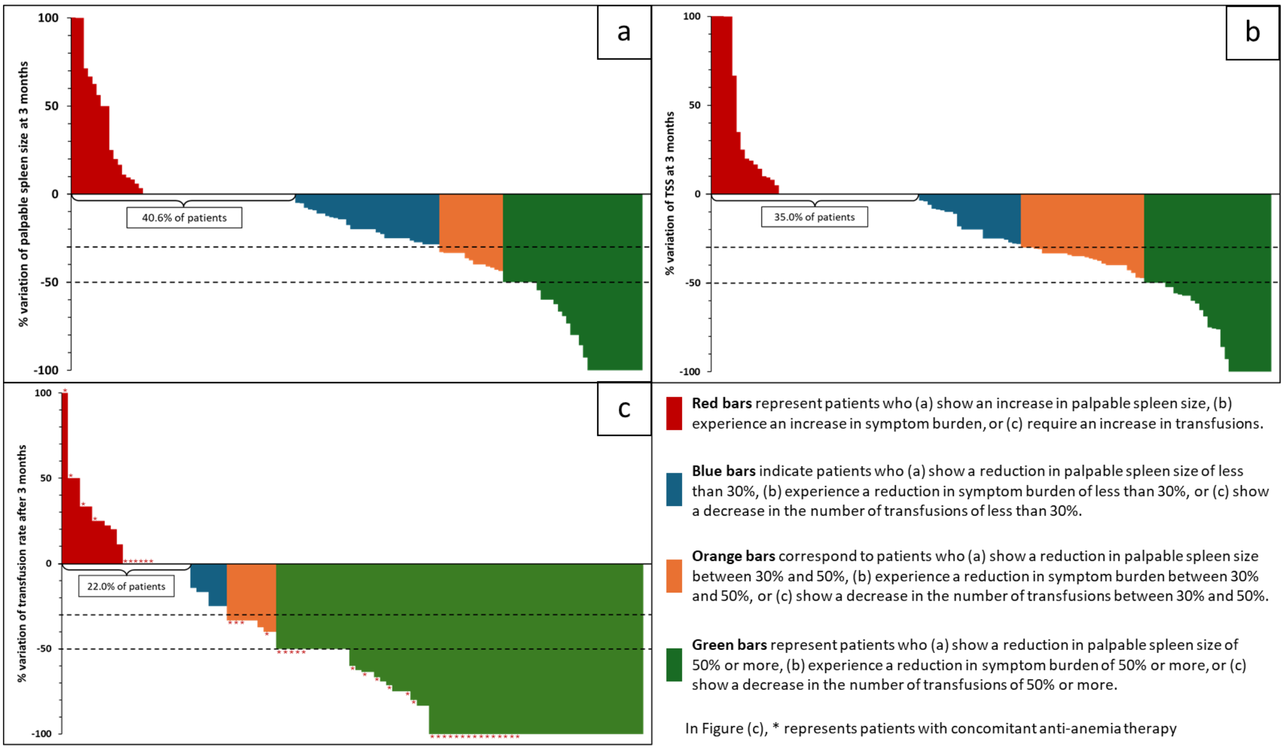

***Supplemental Figure 3**: TSS, Total Symptoms Score*

Supplemental Figure 3: Baseline predictors of IWG-MRT spleen response at 3 and/or 6 months

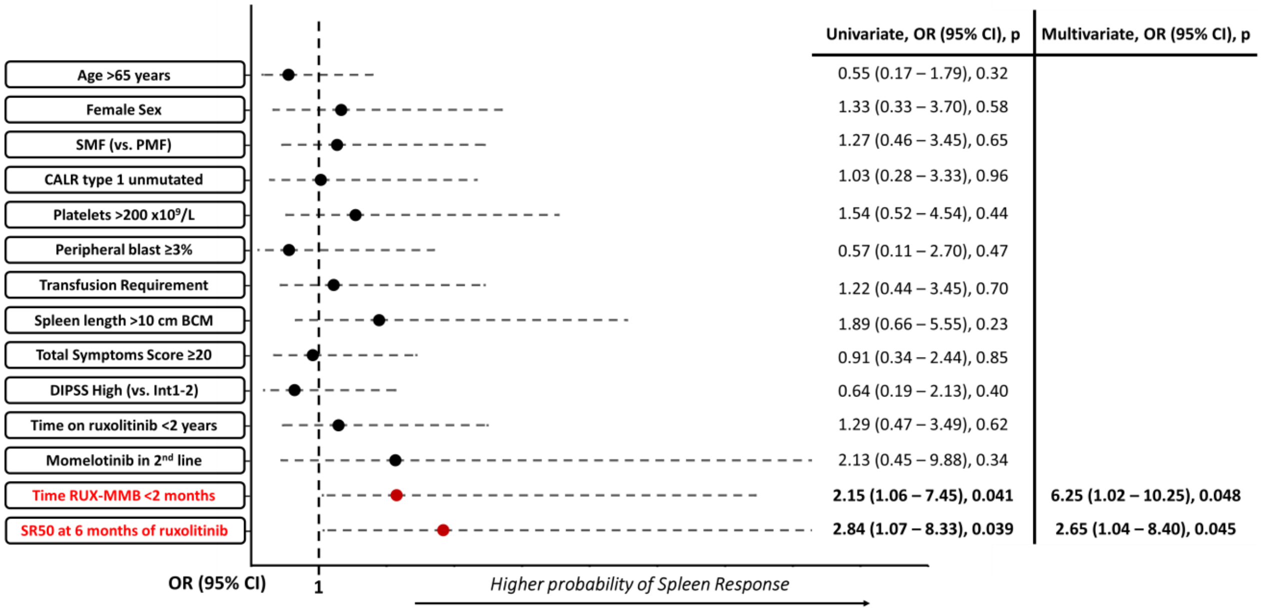

Supplemental Figure 3: *SMF, secondary myelofibrosis; PMF, primary myelofibrosis; BCM, below costal margin; DIPSS, Dynamic International Prognostic Scoring System; Int, Intermediate; RUX, ruxolitinib; MMB, momelotinib; SR50, spleen length reduction of at least 50%.*

Supplemental Figure 4: Progression-Free Survival by combined improvement in anemia and spleen size at 3 months (a) and 6 months (b)

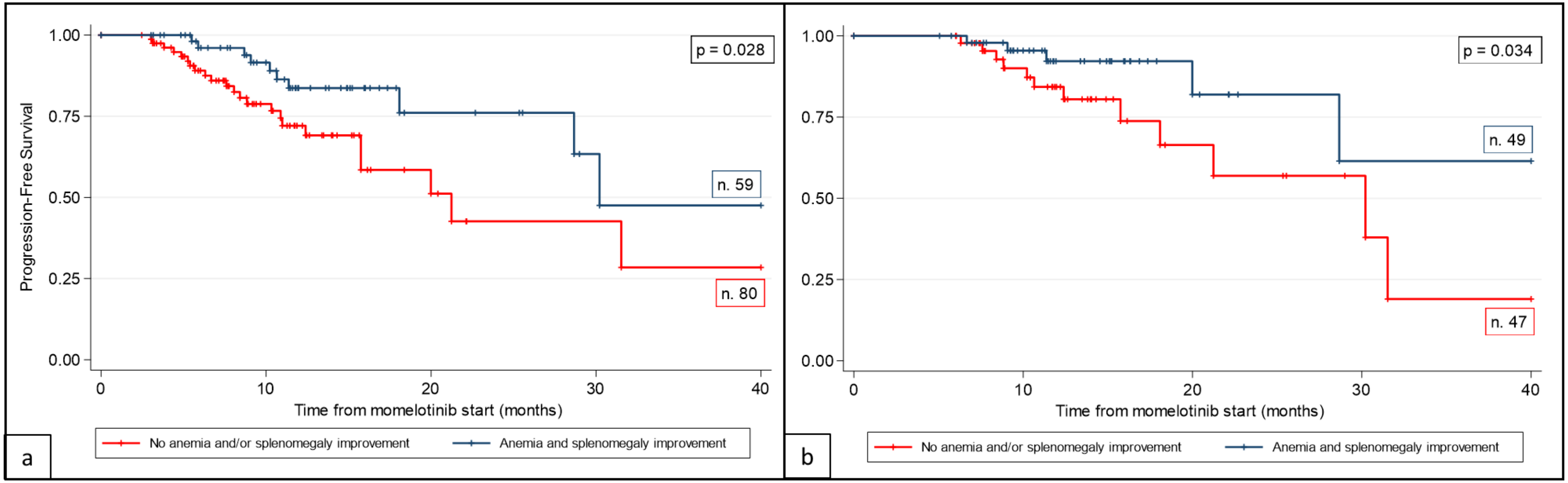

Supplement: Supplementary file 1 — Supplementary Material [file CNCR-132-e70457-s001.pdf]
